# Evolution and biogeography of apple stem grooving virus

**DOI:** 10.1186/s12985-023-02075-2

**Published:** 2023-05-26

**Authors:** Shohreh Shokri, Kamal Shujaei, Adrian J. Gibbs, Mohammad Hajizadeh

**Affiliations:** 1 Department of Plant Protection, Faculty of Agriculture, University of Kurdistan, Sanandaj, Iran; 2 Emeritus Faculty, Australian National University, Canberra, Australia

**Keywords:** *Apple stem grooving virus*, Genome phylogeny, Biogeography, Population Genetics

## Abstract

**Background:**

Apple stem grooving virus (ASGV) has a wide host range, notably including apples, pears, prunes and citrus. It is found worldwide.

**Method:**

In this study, two near complete genomes, and seven coat protein (CP) sequences of Iranian isolates from apple were determined. Sequences added from GenBank provided alignments of 120 genomic sequences (54 of which were recombinant), and 276 coat protein genes (none of them recombinant).

**Result:**

The non-recombinant genomes gave a well supported phylogeny with isolates from diverse hosts in China forming the base of the phylogeny, and a monophyletic clade of at least seven clusters of isolates from around the world with no host or provenace groupings among them, and all but one including isolates from China. The six regions of the ASGV genome (five in one frame, one − 2 overlapping) gave significantly correlated phylogenies, but individually had less statistical support. The largest cluster of isolates contained those from Iran and had isolates with worldwide provenances, and came from a wide range of mono- and dicotyledonous hosts. Population genetic comparisons of the six regions of the ASGV genome showed that four were under strong negative selection, but two of unknown function were under positive selection.

**Conclusion:**

ASGV most likely originated and spread in East Asia in one or more of various plant species, but not in Eurasia; the ASGV population of China had the greatest overall nucleotide diversity and largest number of segregating sites.

**Supplementary Information:**

The online version contains supplementary material available at 10.1186/s12985-023-02075-2.

## Introduction

*Apple stem grooving virus* is the type species of the genus *Capillovirus*, subfamily *Trivirinae*, family *Betaflexiviridae*, and order *Tymovirales* [1]. Apple stem grooving virus (ASGV) has a wide host range [2], but has been most often isolated from rosaceous fruit trees such as apple, apricot, cherry and pear, causing a latent infection in most commercial cultivars but some apple cultivars may develop severe symptoms such as xylem pitting and grooving, phloem necrosis, decreased trunk diameter and the complete decay of the tree thus greatly decreasing its productivity [3, 4]. ASGV is also an important pathogen of many varieties of citrus, causing the tatter leaf disease [5].

Like all capilloviruses, ASGV has filamentous virions, each composed of a positive-sense, single-stranded RNA genome of about 6500 nucleotides excluding the non-stranslated 5’ and the 3’ polyadenylated tail [6, 7] enclosed in a helically constructed tube of c. 1300 subunits of a single species of coat protein (CP). The genome of ASGV has two ORFs [8, 9]. ORF1 covers most of the genome, except for the termini, and encodes a large replicase polyprotein (241 kDa), and the 3’ terminal CP (27 kDa), which is expressed by a sub-genomic RNA [10]. A search of the Pfam database with the replicase polyprotein sequence shows that it encodes, from N- to C-terminus, a viral methyltransferase (v-Mtase), a papain-like protease, a viral superfamily 1 helicase and a RNA-depemdent RNA-polymerase (RdRP). It has two very variable regions [9, 11]; a small one around nts 1590–1710 between the v-Mtase and helicase, and and a much larger one around nts 4749–5604 between the RdRP and the CP domains; the amino acid sequences encoded by the two variable regions of ORF1 only match ASGV sequences in BLASTn or BLASTp searches of the GenBank database, and their functions and/or the reasons for their variability are unknown. The ORF2 is in the − 2 reading frame of the second variable region, and encodes a movement protein (MP) of 36-kDa [5, 12] of the ‘30 kDa superfamily’. Thus, in summary, the genome has five regions; region 1 encodes a v-Mtase, the short region 2 is variable and of unknown function, region 3 encodes a papain-like endopeptidase, a helicase and a RdRP, region 4a is the second variable region and encodes a “polyprotein linker domain” [13], which completely overlaps region 4b in the − 2 reading frame, and ORF 1 is completed by region 5, which is the CP gene.

Although several papers have reported analyses of gene sequences of regional populations of ASGV [3, 14–16], we know of no reports describing the global population structure of this virus. Currently (November, 2022) 123 full length ASGV genomic sequences, together with the sequences of the CP genes of over 300 isolates are available from the GenBank database.

Here we report the CP gene sequences of seven Iranian ASGV isolates, and the full length genomic sequences of two other isolates, and place these within phylogenies of all other reported ASGV isolates hoping to obtain useful insights to the origins, phylogeny, population evolution and biogeography of the virus. Iranian isolates are of particular interest in the search for the origins of ASGV as Iran is at the western end of the legendary “Silk Road”, which from around 200 BCE to 1800 CE connected traders of Eurasia to those of China and east Asia, and which is bounded in central Asia to the north by the Tien Shan mountains, which are considered the site of domestication of apples [17–19], if therefore ASGV had first infected apples while they were being domesticated, then isolates from Iran should be basal in the ASGV phylogeny.

## Materials and methods

### Samples

As ASGV does not induce visible disease symptoms in infected trees and fruits of all commercial cultivars [20], we collected 174 leaf samples of symptomatic and non-symptomatic apple, apricot and pear trees from several locations in the west and northwest of Iran during the spring and summer of 2017–2020.

### Total RNA extraction, cDNA synthesis and RT-PCR

Total RNA was extracted from each leaf sample using the method described by [21] with minor modifications. Reverse transcription (RT) reactions were done using a cDNA solution synthesis kit (HyperScriptTM Reverse Transcriptase, GeneAll, South Korea) and random hexamer primers. PCR reactions done using specific primers, ASGV-U and ASGV-2 [22] in the PCR master mix (GeneAll, South Korea). PCR products were electrophoresed in a 1.2% (w/v) agarose gel (containing safe stain) in 1X TAE buffer.

### Amplify Full-CP gene and complete Coding Region

The complete CP genes of seven isolates (Table S1), obtained from five apple trees, and one apricot and one pear infected by ASGV were amplified using the specific primer pairs, ASGV-MF and ASGV-MR (Table 1). The complete genomes of two other isolates, K from the west and the Mn from northeast of Iran (Table S1), were amplified using six pairs of primers (Table 1). The PCR products were ligated into the pTG19 cloning vector (SinaClon, Iran), and the ligation mix was used to transform *Escherichia coli* strain DH5α as described by Chung et al. [23]. The identities of the recombinant plasmids were confirmed by restriction analysis and a purified clone from each isolate was sequenced by Macrogen Inc. (Seoul, South Korea).

**Table 1:** List of primers used in this study

Primer name	Sequence 5’-3’	Product size (bp)	Position	Reference
ASGV-U	CCCGCTGTTGGATTTGATACACCTC	499	5873–5897	[22]
ASGV-2	GGAATTTCACACAGACTCCTAACCCTCC		6345–6371	
ASG-1 F	TCAAATGGCTTTCACTTACAG	1154	39–58	This study
ASG-1R	GAATTTGTCTTTCACCCATTG		1176–193	
ASG-2 F	GCATTTCAAAAATTTTGGRATGAG	1099	1127–1144	This study
ASG-2R	CAAACTTTCCAAGRTCRAACA		2206–2226	
ASG-3 F	GAAGCGCAAATGTCAAATGA	1260	2166–2185	This study
ASG-3R2	ACCGGTATATGGCATTTGAACCA		3406–3426	
ASG-4F2	TTATTGAACCAGTTGAGTGGTT	1255	3404–3421	This study
ASG-4R	GCCTCTCACCTAATCTGTAG		4640–4659	
ASG-5 F	TACAGATTAGGTGAGAGGCT	1031	4641–4660	This study
ASG-5R	TTGTTGAAGCACGTCTTCC		5654–5672	
ASGV-MF	ATGAGTTTGGAAGACGTGCTTC	714	5646–5667	This study
ASGV-MR	AACCCCTTTTTGTCCTTCAGTACGAA		6292–6316	

### Global ASGV sequence analysis

One-hundred and twenty-five full length ASGV genomic sequences (SDFile 1) were found in GenBank (November 2022) and downloaded. The 5’ and 3’ terminal non-coding regions were removed, and the aligned ORFs checked for recombination using RDP 4.95 [24]. The 54 recombinant (rec) sequences and five duplicate sequences were removed from further analyses. The relationships, genetic diversity and phylogenetic information of the remaining 66 genomic sequences, and each of their six genomic regions, were compared in various ways. First their nucleotide sequences and the amino acid sequences they encoded were aligned using the RevTrans server [25], and used to generate ML phylogenetic trees using the maximum likelihood method PhyML [26] with the Shimodaira-Hasegawa test [27] for node support, and by the neighbor-joining (NJ) method in ClustalX [28] with bootstrapping to assess node support. The patristic distances within each of these trees were compared pairwise using PATRISTIC [29]. A BLAST search of the GenBank database using the ASGV Reference sequence (NC_001749) and the Entrez search term “NOT apple stem grooving virus” found the most closely related sequences to be those of Yacon virus A (NC_030657; YVA), *Breadfruit capillovirus* 1 (MW328738; BCV1), and *Polyscias capillovirus* 1 (ON240065; PCV1). The YVA, BCV1 and PCV1 sequences were therefore used to root the ASGV phylogenies.

Programs in DnaSP v.6.10.01 [30] were used to estimate average pairwise nt diversity (π), mean synonymous substitutions per synonymous site (dS), mean non-synonymous substitutions per non-synonymous site (dN) and ratio of non-synonymous nt diversity to synonymous nt diversity (dN/dS). It was concluded that genes were under positive, neutral or negative selection when their dN/dS ratios were > 1, =1 and < 1, respectively. Tajima’s D statistical test was used to identify non-random evolutionary events such as population expansion, bottlenecks and selection by comparing the estimated number of segregating sites with the mean pairwise difference among sequences [31]. The five gene regions were also checked for positive selection using the Fast Unconstrained Baysian AppRoximation (FUBAR) and Fixed effect Likelihood (FEL) in the Datamonkey programs [32].

Then we downloaded from GenBank (November 2022) the CP gene sequences of more than 320 isolates from 12 countries: Brazil, China, Czech Republic, Germany, India, Iran, Japan, Serbia, South Korea, Taiwan, Turkey, the USA. To these we added the newly determined CP sequences of seven ASGV isolates and two CP sequences of K and Mn isolates. Duplicate sequences were deleted leaving 276 sequences. The ASGV CP sequences were aligned using Clustal W [33] in the MEGA X program [34] or using RevTrans [25] with default parameters, and also used to generate ML phylogenetic trees using PhyML [26]. The patristic distances within each of these trees were compared pairwise using PATRISTIC [29]. The extent of genetic differentiation and gene flow (F_ST_) were checked using the DnaSP 5.10 program [30]; values of F_ST_ > 0.25 indicate that there are large genetic differences between the subpopulations, and they have probably not been genetically connected, whereas if < 0.25 then gene flow may have occurred.

To check whether the Introduction (above) was appropriately focussed for introducing our study we summarized it using the artificial intelligence online “ChatGPT: Optimizing Langauge Models for Dialogue” site (https://openai.com/blog/chatgpt/).

## Results

### New Iranian isolates

ASGV was detected in 18 (10.3%) of samples in both west and north west Iran (Table S1). All nine full-CP sequences contained open reading frames (ORFs) 714 nt in length with 237 deduced amino acid residues. ASGV has the codon UAG as stop codon in all sequences. The greatest nucleotide diversity was at position 590, and the least diversity was in their 5′-terminal regions. The encoded amino acid sequences, showed only two differences, at positions 52 and 122, and the C and N-terminal regions were completely conserved. Overall mean distance between our sequences was 0.009 ± 0.002 and sequence identity among isolates from different hosts and location in our study ranged from 98.6 to 100% and 99.2 to 100% in the nucleotide (nt) and amino acid (aa) sequences, respectively. No differences were found between the encoded CP sequences of the Iranian isolates and those of the most closely related isolates from other countries except for one aa in isolate NG where Proline replaced Leucine at position 122. The sequence of the CP gene of KS3 (isolated from apple), KZ (apricot), D3 (apple), NG (pear), SS (apple), SS4 (apple), X4 (apple) were deposited in GenBank with Accession Codes MK354030 - MK354036, respectively. Two sequences, SS4 and KS3, had 100% nt identity, and therefore their aa sequences were identical, so KS3 was removed from further analysis. Finally, two isolates from different apple cultivars, which were geographically most separate, K from Sanandaj in Kurdistan Province isolated from Red Delicious and Mn from Marand in east Azerbaijan Province and also isolated from Golden Delicious, were selected for the sequencing of their complete coding regions. Their sequences were found to be the same length, and had 96.4% nucleotide identity. They were deposited in GenBank with Accession Codes OQ263370 (isolate MN18) and OQ263371 (isolate K). The greatest and smallest identities in separated ORFs were found in the RdRP polyprotein and CP respectively, in both nucleotide and amino acid comparisons.

### Recombination

When the complete genomic sequences were checked for phylogenetic anomalies using the Recombination Detection Program Version. 4 (RDP4), 54 of the sequences (43 of them from China, four from Canada, three from India, two from Brazil, one from either South Korea and Taiwan) were found to have rec regions (Table S2). These were removed from the alignment used for phylogenetic and population genetic analysis because rec sequences distort the results of most algorithms used for reconstructing phylogenies. The CP genes were also checked by RDP4, but no additional rec sequences were found.

### Phylogeny

**Fig. 1:**
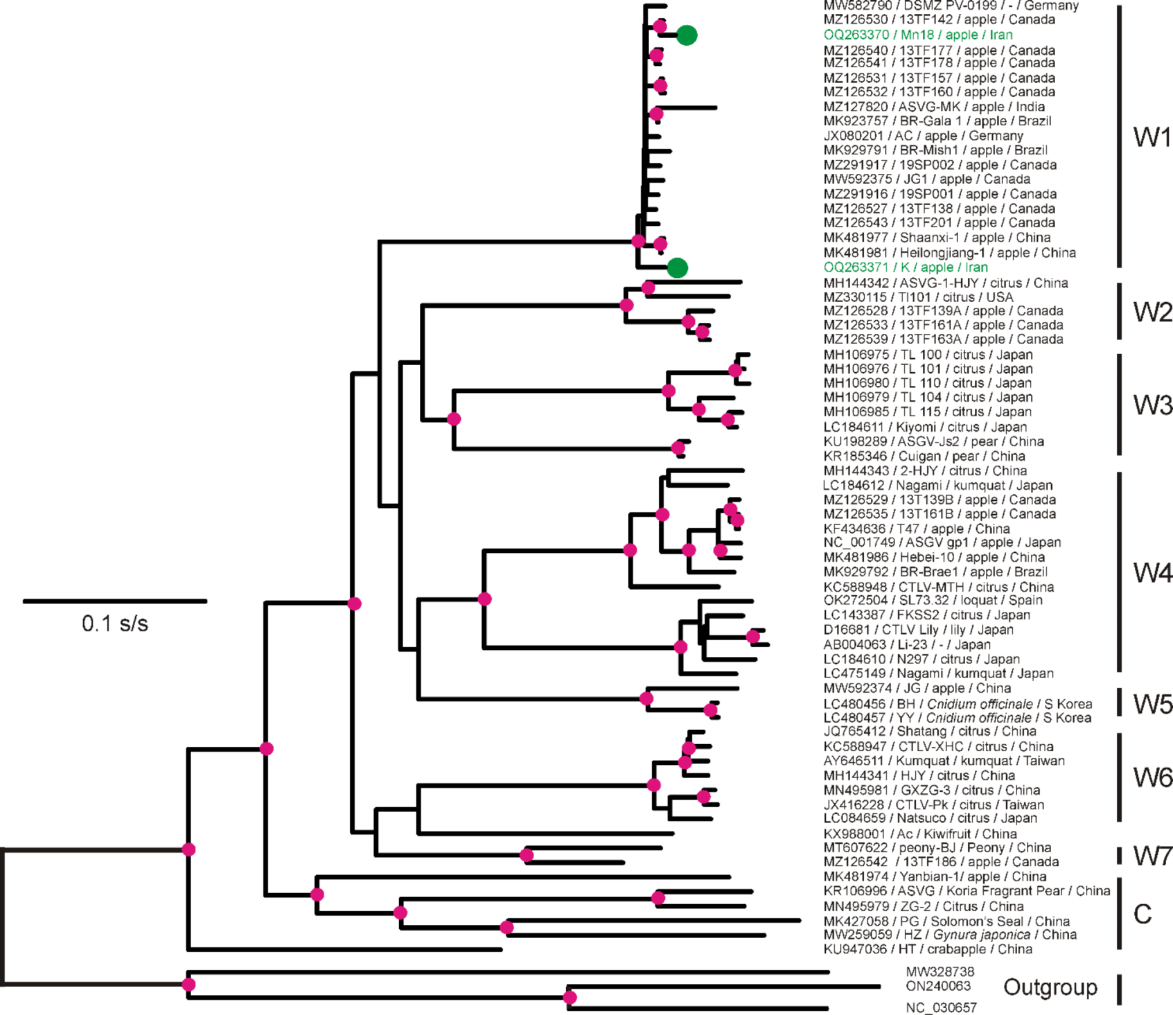
A phylogeny of 66 non-recombinant ASGV isolates and an outgroup of three other capilloviruses (Yacon virus A, NC_0303657; breadfruit capillovirus 1, MW328738; and Polyscias capillovirus 1, ON240065); the branch to Yacon virus A is drawn 5% of its calculated length, and those of the other two half their calculated length. The two Iranian isolates are marked in green. Significant clusters are marked C (basal cluster of isolates from China) and W1-W7 (world clusters). This phylogeny was calculated using PhyML (GTR + γ + I) from the aligned non-recombinant genomes. All nodes that had statistical support greater than SH 0.9 are marked with a red disc. The scale bar represents 0.1 substitutions per site

The root of the ASGV phylogeny was established using sequences of one or more of three other capilloviruses as outliers, and all those sequences, whether used singly or in combinations, formed a single branch to the ASGV part of the combined phylogeny. Phylogenies were calculated from the aligned 66 complete concatenates using either their nts or encoded aa sequences, and using either the PhyML or NJtree methods. The four combinations gave, in essence, the same statistically supported phylogeny with no consistent statistically significant differences; Fig. 1 shows that calculated from the nucleotide sequences by the ML algorithm. All the basal region of the ASGV phylogeny (i.e. that closest to the outgroup) only involved isolates from China; one basal branch was to a single sequence (KU947036), the other to a cluster of five isolates from China, and from diverse hosts, both mono- and dicotyledonous (see Discussion). The cluster of five Chinese isolates was paraphyletic to a monophyly of, at least, seven clusters, each of two to 19 isolates, seven of which included isolates from China (Fig. 1); the two new Iranian sequences were members of the largest cluster W1. All 19 isolates of the W1 cluster with recorded hosts came from apple, and were collected from plants in all parts of the New and Old World (Fig. 1).

The GenBank database provided additional CP sequences of ASGV, however phylogenies calculated from all 276 had less statistical support than those calculated from the complete genomes, as would be expected as they are only 10% of their length. Few clusters in the CP phylogeny had more than SH 0.7 support and the basal cluster of Chinese isolates was not formed and its component sequences were attached as outliers to other clusters. All the other clusters (Fig. 1) remained intact with added CP sequences, except cluster W4, which separated into the two sub-clusters.

The largest CP cluster was the enlarged, but still compact, Cluster W1, which contained the new Iranian isolates and had a basal node with support of SH 0.82. This CP cluster consisted of 69 isolates, 52 of them from apple, however others in the cluster were isolated from a wide variety of hosts [2] in surveys of Himachal Pradesh, northern India (see Discussion). The W1 isolates were from countries of both the Old World (China, India, South Korea), especially Eurasia (Czechia, Germany, Iran, Serbia and Turkey), and also the Americas (Brazil and Canada).

### Genetic diversity

The relationships between the six genomic regions was compared by population genetic analysis (Table 2). This showed that in all estimated features, the 1, 3, 4b and 5 regions were closely similar, and significantly different from those of regions 2 and 4a. In particular, the dN/dS ratio, which is a measure of selection, indicates that regions 1, 3, 4b and 5 are all under strong negative selection (dN/dS range 0.042–0.065), whereas those of regions 2 and 4a (dN/dS 0.546 and 3.117) respectively, may be under positive selection. The Tajima’s D metrics for most regions are similar, but those of region 2 (variable) and region 5 (the CP gene) differ from the others and are negative (i.e. indicative of a population expansion after a bottleneck), although these values are not statistically significant.

**Table 2:** Genetic diversity of 66 non-recombinant ASGV isolates in different regions of the genome

Regions^a^	Length (nt)	ϴ^b^	π	d_N_	d_S_	d_N_/d_S_	Tajima’s D
1	1545	0.494	0.171	0.041	0.636	0.065	0.070^ns^
2	171	0.729	0.464	0.397	0.728	0.546	-0.257^ns^
3	3033	0.499	0.181	0.039	0.702	0.056	0.177^ns^
4a	855	0.454	0.144	0.169	0.054	3.117	0.205^ns^
4b (over-lapping − 2)	960	0.435	0.133	0.021	0.504	0.042	0.106^ns^
5	711	0.354	0.079	0.018	0.288	0.062	-0.593^ns^

^a^ ASGV genomic regions analyzed: 1, the methyltransferase domain of the RNA-dependent RNA polymerase (RdRP); 2, located within the RdRP; 3, the P-pro, Hel and RdRP domains of ORF1; 4, located within the movement protein (MP) and 5; the coat protein region.

^b^ Proportion of segregating sites.

Nonetheless the topology of the ML phylogenies of the six regions was closely similar and, when these topologies were compared pairwise using PATRISTIC, the correlations ranged from a maximum of 0.966 (regions 1 versus 3) to 0.497 (regions 2 versus 5), with mean correlations for regions 1, 2, 3, 4a, 4b and 5 of 0.861, 0.712, 0.794, 0.768 and 0.779 respectively (p < 0.00001).

For the ASGV CP, nucleotide diversity (π) was estimated as the mean nucleotide distance between sequence pairs using the Mega X Program. The global nucleotide diversity of ASGV CP was 0.084 ± 0.006, same to the nucleotide diversity of the CP gene of cherry virus A (0.084) [35], another *Capillovirus*, but with a smaller genome than other viruses in the family *Betaflexiviridae* [36, 37].

The pattern of divergence of the ASGV population was poorly resolved phylogenetically, perhaps because this is a recently diverged population. To test this possibility, we compared the genetic structure of the ASGV CP populations in the different world regions, grouping the isolates into the five main subpopulations: India, East Asia (China, Japan, South Korea, Taiwan), Middle East (Iran and Turkey), Europe (Czech Republic, Germany and Serbia) and the Americas (Brazil, Canada and the USA). All geographic subpopulations showed small nucleotide diversity values (0.068, 0.083, 0.024, 0.040, and 0.072, respectively; Table 3) that were in the same range as those found between the phylogenetic subpopulations (0.034 to 0.092).

Evolutionary events such as population expansion, bottlenecks, and selection, may be tested by Tajima’s D [31] and by Fu & Li’s D and F static tests [38] using the DnaSP 5.10 program. The significantly negative values of Tajima’s D and Fu and Li’s F* metrics for the Middle East population and Fu and Li’s F* metrics for the East Asia population (Table 3) are evidence of recent population expansion of that populations, however, the statistics of similar past population expansions of the Indian, Europe and the Americas were indicative but not statistically significant.

**Table 3:** Genetic diversity of coat protein gene of ASGV in different geographic area

Populations	N	S	π	d_N_	d_S_	d_N_/d_S_	Tajima’s D	Fu & Li’s D*	Fu & Li’s F*
India	55	210	0.068	0.017	0.243	0.058	-0.298^ns^	-1.067^ns^	-0.922^ns^
East Asia	169	369	0.083	0.020	0.302	0.054	-1.065^ns^	-2.735*	-2.296^ns^
Europe	14	102	0.040	0.008	0.147	0.052	-0.694^ns^	0.105^ns^	-0.132^ns^
Middle East	12	80	0.024	0.002	0.097	0.024	-1.790*	-2.219*	-2.402*
Americas	26	170	0.072	0.013	0.275	0.039	-0.159^ns^	-0.048^ns^	-0.099^ns^
Total	276	413	0.084	0.019	0.309	0.062	-1.172^ns^	-4.039**	-2.999**

N: number of isolates, S: number of polymorphic (segregating) sites, π: nucleotide diversity, d_N_: non-synonymous nucleotide diversity, d_S_: synonymous nucleotide diversity, ns: non significant, Tajima’s D: comparing the estimated number of segregating sites with the mean pairwise difference among sequences.

### Codon Selection

The FUBAR method detected positively selected codons in regions 2, 4a, 4b and 5 respectively, but none in regions 1, and 3 (Table 4), and most of these sites were also detected by the FEL method (P > 0.05). Unexpectedly, one site of region 2 and three sites of region 4a were found to be under strong negative selection by both methods.

**Table 4:** Estimates of selection pressures acting on the codons of five genome regions of isolates of ASGV using FUBAR and FEL methods in Datamonkey

Regions	Number of sites	Codons under PS^a^ (FUBAR)	Number of NS^b^ (FUBAR)	Codons under PS (FEL)	Number of NS (FEL)
1	515	0	487	0	466
2	57	**38**^**c**^, **44**	19	**38, 44**	19
3	1011	0	977	0	924
4a	286	204^d^	5	189^d^	5
4b	320	**296, 304, 307, 308, 314**	266	**296**, 302, **304, 307, 308, 314**	257
5	237	**27, 94, 98**, 103	137	**27, 94, 98, 117, 137**	125

^a^PS: Positive selection, ^b^NS: Negative selection, ^c^codons identified by both FUBAR and FEL, ^d^ Number of sites under positive selection

**Fig. 2:**
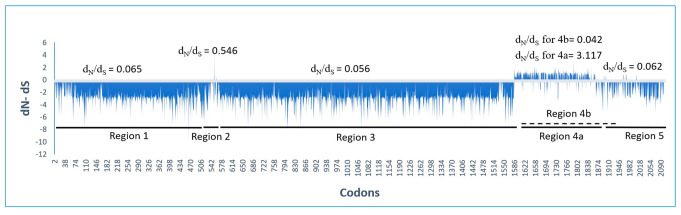
Selective pressure on different regions of apple stem grooving virus

As described above the selection of codons in different regions of the genome, was measured by dN/dS estimates (Table 2) and by FUBAR and FEL estimates (Table 4). The relationship between these measures was clarified by plotting the FUBAR estimates for individual codons in all regions of the genome, and comparing these estimates with the dN/dS estimates (Fig. 2). It can be seen that the codon by codon FUBAR estimates correlate with the dN/dS estimates, however the individual codons that were found by FUBAR to be positively or negatively selected (Table 4), as judged statistically, are few and not indicative of the regional results. Interestingly, there is a patch of indels around 530–620 and then again between 1588 and 1865, corresponded to regions 2 and 4a between consensus sequences of lineages A and B.

Strong selective constraints on a gene/domain are likely to indicate that it has a key role in viral functions. In addition, the presence of positively selected sites in a gene, however few, suggests that host-associated selection probably is a significant factor in the evolution of the virus, possibly its host range. Regions 2, and 4a are under strong positive selection, and only 33.3% and 1% of their codons were found be under negative selection. Interestingly, in region 2, 70.3% and 64.3% of the codons were found be under strong positive selection, based on the FUBAR and FEL methods, respectively. This region encodes a “polyprotein linker domain” [13] that only matches ASGV sequences in BLASTp searches, and its role in ASGV is unknown. The role of the positively selected amino acids is unknown, but might be revealed by sequencing the genomes of isolates adapted to different host species.

## Discussion

The objectives of this investigation were to better understand the evolution, phylogeny, and genetic structure of the world ASGV population, and the place of the Iranian population in it, using a variety of different approaches. The near complete genomic sequences of two new Iranian ASGV isolates are reported, and together with those downloaded from GenBank show that non-rec genomic sequences form a well-supported phylogeny. To check whether some regions of the ASGV genome gave different, or better supported, phylogenetic information than others, we analysed and compared the six distinct genomic regions of the non-rec ASGV genomes. We confirmed earlier reports that the different regions of the ASGV genome have evolved at different rates, but found that nonetheless those regions have evolved in parallel, and therefore a phylogeny of complete genomic sequences is more informative than phylogenies of any one of the genomic regions, or combination of regions. These comparisons also showed that phylogenies of the CP region can only be used safely to extend the genomic clusters as their statistical support is less, and inadequate to distinguish the basal regions of the phylogeny.

The phylogeny of complete non-rec ASGV genomes showed a statistically supported basal group of six isolates from China, confirming the phylogeny of Tan et al., [39], and suggesting that South East or East Asia, which is the centre of domestication of citrus and relatives [40], may be the ‘centre of emergence’ of ASGV, not the Tien Shan mountains of central Asia where the early stages of apple domestication from *Malus sieversii* occurred [18, 19]. Further support for this conclusion comes from the population genetic structure of the ASGV population in East Asia when compared to other populations; it had the greatest overall nucleotide diversity (π = 0.083), and largest number of segregating sites (S = 369), and dN/dS of 0.054 (Table 3), suggesting it is the oldest. By contrast the Iranian population of ASGV resembled those of other non-East Asian regions of the world. In addition, the indels of regions 2 and 4b contributed notable to the differences between lineage A and B sequences.

Three quarters of the complete ASGV sequences, we analyzed were from isolates from apples, and most of the others were from citrus, and the hosts from which CP sequences were obtained were also dominated by Citrus, Malus, Prunus and Pyrus spp. However, the isolates of Cluster C came from *Gynura japonica* (a medicinal herb from the *Asteraceae*), *Polygonatum kingianum* (a monocotyledonous garden plant), citrus and pear, both dicotyledonous asterid plants, apple, and crab apple (*Malus sylvestris*). Furthermore, a survey of a wide range of plant species in Himachal Pradesh, northern India [2] isolated ASGV from a wide range of species including two species of bamboo (*Dendrocalamus* spp. and *Fargesia somnigensis*). Thus it is likely that ASGV is not just a ‘fruit tree virus’ but has a wider host range among monocotyledonous and dicotyledonous plants, and testing of plants from basal angiosperm groups, or their SRA data, may identify other hosts.

## Conclusion

The phylogeny of complete non-rec ASGV genomes showed a statistically supported basal group of isolates from China, confirming that East Asia, which is the centre of domestication of citrus and relatives, may be the ‘centre of emergence’ of ASGV. Further support for this conclusion comes from the population genetic structure of the ASGV population in China when compared to other populations.

## Electronic supplementary material

Below is the link to the electronic supplementary material.

Supplementary Material 1

## Data Availability

The sequences generated in this study are available at NCBI.

## Abbreviations

Aa: Amino acid
ASGV: *Apple stem grooving virus*
CP: Coat Protein
FEL: Fixed effect Likelihood
F_ST_: Gene flow
FUBAR: Fast Unconstrained Baysian AppRoximation
MP: Movement Protein
ML: Maximum Likelihood
NJ: neighbor-joining
Nt: Nucleotide
ORF: open reading frames
RDP4: Recombination Detection Program Version. 4
RdRP: RNA-depended RNA-polymerase
Rec: Recombinant
RT: Reverse transcription
SH: Shimodaira-Hasegawa
v-Mtase: viral methyltransferase
YVA: *Yacon virus A*
